# Reliability and validity of the adapted Finnish version of the early onset scoliosis questionnaire (EOSQ-24)

**DOI:** 10.1007/s43390-024-00861-8

**Published:** 2024-04-05

**Authors:** Hermanni Haapala, Anne Salonen, Eetu Suominen, Johanna Syvänen, Jussi Repo, Hiroko Matsumoto, Matti Ahonen, Ilkka Helenius, Antti Saarinen

**Affiliations:** 1 Department of Paediatric Surgery, Orthopaedics and Traumatology, New Children’s Hospital, Helsinki, Finland; 2 Department of Paediatric Surgery, Tampere University Hospital, Tampere, Finland; 3 Department of Paediatric Surgery, Orthopaedics and Traumatology, University of Turku and Turku University Hospital, Turku, Finland; 4 Department of Orthopaedics and Traumatology, University of Tampere and Tampere University Hospital, Tampere, Finland; 5 Department of Orthopaedic Surgery, Harvard Medical School, Boston, MA USA; 6 Department of Orthopedic Surgery and Sports Medicine, Boston Children’s Hospital, Boston, MA USA; 7 Department of Orthopaedics and Traumatology, University of Helsinki and Helsinki University Hospital, Helsinki, Finland; 8 Department of Surgery, Jyväskylä Central Hospital, Jyväskylä, Finland

**Keywords:** Early onset scoliosis, Psychometrics, Validation, EOSQ-24

## Abstract

**Background:**

EOSQ-24 is a disease specific patient-reported outcome score used to assess the quality of life in patients with early-onset scoliosis. The aim of this study was to translate and cross-culturally adapt the English version of the EOSQ-24 to Finnish language and to assess the reliability and validity of the translation.

**Methods:**

Cross-cultural adaptation and cross-cultural validation were performed to the Finnish translation of the EOSQ-24. Patients and/or their caretakers were then recruited to assess the psychometric properties of the translation. We assessed the internal consistency, test–retest reliability, floor and ceiling effects, and discriminative abilities. One-hundred-and-three patients filled the questionnaire.

**Results:**

EOSQ-24 was successfully translated into Finnish. The translation showed excellent internal consistency (Cronbach alpha 0.94), satisfactory item-total correlations ranging from 0.6 to 0.9, and moderate to strong inter item correlations. Test–retest reliability ranged from 0.7 to 0.96 indicating good to excellent agreement. Patients with neuromuscular and syndromic scoliosis reported lower EOSQ-24 scores when compared to patients’ idiopathic and congenital scoliosis. There was a significant negative correlation between major curve and EOSQ-24 scores in patients with idiopathic early onset scoliosis.

**Conclusion:**

The internal consistency and test–retest reliability of the measure were found to be satisfactory. A marked ceiling effect was observed, indicating a potential source of error.

## Introduction

Early Onset Scoliosis (EOS) is defined as scoliosis diagnosed before the age of 10 years. EOS can progress to a severe deformity and may compromise the normal development of lungs and thorax, lead to the development of thoracic insufficiency syndrome and cause significant morbidity and mortality if left untreated [1, 2]. EOS is a heterogenous condition and may be defined as syndromic, neuromuscular, congenital, or idiopathic etiology [3].

The treatment of EOS aims to prevent the progression of the deformity while allowing spinal growth. Conservative treatment is used in mild deformities and to delay the surgical treatment by slowing the progression of more severe deformities. Surgical treatment is usually indicated when the progressive deformity reaches 45 degrees [4]. Growth-friendly treatment may be performed with surgically or magnetically controlled growing rods, vertical expandable prosthetic titanium ribs (VEPTR), or growth guidance implants. Magnetically lengthened growing rods have decreased the need for surgical procedures and revision surgeries [5, 6].

Early Onset Scoliosis Questionnaire 24 (EOSQ-24) is a disease specific health-related outcome measure developed for assessing the patient and caretaker quality of life (HRQL) [7]. Etiology of the EOS is significantly associated with the quality of life, with syndromic and neuromuscular scoliosis having lower EOSQ-24 scores when compared to congenital and idiopathic scoliosis [8, 9]. Previous studies have demonstrated good internal consistency, test–retest reliability, and responsiveness of the EOSQ-24 questionnaire [7, 10–21].

The objective of this study was to translate and transculturally adapt the English version of the EOSQ-24 into Finnish, and to assess the validity and the reliability of the Finnish version.

## Methods

EOSQ-24 consists of 24 question items, which can be answered by the patient or the caretaker. These items are then transformed into 4 domains Health-Related Quality of Life, Parental Burden, Financial Burden, and Satisfaction. The Health-Related Quality of Life domain is further categorized into 8 sub-domains: General Health, Pain and Discomfort, Pulmonary Function, Transfer, Physical Function, Daily Living, Fatigue, Emotion [7].

The English version of the EOSQ-24 was translated into Finnish by two orthopedic surgeons fluent in both Finnish and English. A reconciliation version was produced by the two translators by comparing the translations with each other’s and the original English version. The reconciliation of the Finnish translation was then translated back to English and compared to the original EOSQ-24 in a back translation review. The reconciliation version was thereafter harmonized comparing the results of the translation process. The final Finnish translation was reviewed by an expert committee familiar with EOSQ-24. Cognitive debriefing was conducted, in which the translation was tested with patients and caretakers and found to be easily understandable and relevant. The prefinal version was produced based on the results of the cognitive debriefing and pretesting results. The prefinal version was then proofread by a professional linguistics expert, after which the final version was produced.

EOSQ-24 was collected from children who visited out-patient clinic for assessment for early onset scoliosis. Caregivers were fluent in Finnish. To assess the test–retest reliability, a second survey was sent 2 weeks after the initial assessment by mail.

The internal consistency was analyzed using the Cronbach α coefficient. Internal consistency over 0.7 was considered as satisfactory [22]. Item-total correlation was tested to assess if the individual items are consistent with the overall test and correlate with the total score. Item-total correlation over 0.3 suggests good consistency between the item and the overall test [23]. Inter item correlation was tested to assess internal consistency, with values 0.2 to 0.8 considered to indicate good consistency [23]. The test–retest reliability was analyzed using the intraclass correlation coefficient (ICC) for the patients who had answered the EOSQ-24 twice. ICC values over 0.40 are considered as satisfactory [24]. Floor and ceiling effects were calculated, and the skewness of the data was analyzed. Discriminative validity for scoliosis etiologies was tested using Kruskal–Wallis test and Dunn’s test. The relation of major curve and EOSQ-24 scores was analyzed using Pearson's correlation. The analysis was stratified by etiology, calculating the correlation coefficient for each etiological group individually.

## Results

The translation of the EOSQ-24 into Finnish was successful. Items were easily translated into the Finnish language. The translation was tested with 20 pediatric patients and caretakers and found to be easily understandable. Back translation and pilot testing resulted in no modifications into the final version.

In all, 103 patients were recruited. From these patients, 74 (72%) answered the follow-up questionnaire. The etiology of the scoliosis was idiopathic in 53 patients, neuromuscular in 25 patients, congenital in 17 patients, and syndromic in eight patients. Of the syndromic patients one had diastrophic dysplasia, one had Down syndrome, one had neurofibromatosis I, and five had unspecified syndrome. Patient characteristics are presented in Table 1.

**Table 1:** Patient characteristics

Age at diagnosis (years)	5.5 (SD 3.3)
Age at survey (years)	9.1 (SD 4.0)
Female	56 (53%)
Etiology	
Congenital	17 (17%)
Neuromuscular	25 (24%)
Idiopathic	53 (51%)
Syndromic	8 (7.8%)
Operative treatment	30 (29%)
Major curve at survey (degrees)	25.7 (SD 2.7)

*SD* standard deviation

The mean score in the General health domain was 70.6 (standard deviation [SD] 18) in the congenital group, 75.7 (SD 21) in the idiopathic group, 57 (SD 24) in the neuromuscular group, and 59.4 (SD 15) in the syndromic group (Kruskal–Wallis test *p* = 0.003). The lowest scoring domains were Daily Living in congenital group (67.7, SD 33), neuromuscular group (34, SD 33), and syndromic group (42.2, SD 39), and general health domain in the idiopathic group.

## Internal consistency

The Cronbach alpha for the 24-question total score was 0.94, indicating excellent reliability. Removing any subdomains did not significantly affect the internal consistency (Table 2). Item-total correlations for the domains ranged from 0.63 (Pain and Discomfort, Pulmonary Function) to 0.89 (Parental Burden). Inter item correlations ranged from 0.4 to 0.8 indicating moderate to strong correlation between the domains.

**Table 2:** Internal consistencies and item-total correlations

Item	Cronbach alpha if item is dropped	Item-total correlation
General health	0.94	0.69
Q1	0.96	0.68
Q2	0.97	0.59
Pain and discomfort	0.94	0.63
Q3	0.96	0.61
Q4	0.96	0.62
Pulmonary function	0.94	0.63
Q5	0.97	0.55
Q6	0.97	0.50
Mobility	0.94	0.77
Q7	0.96	0.81
Physical function	0.94	0.79
Q8	0.96	0.76
Q9	0.96	0.74
Q10	0.96	0.81
Daily living	0.94	0.80
Q11	0.96	0.81
Q12	0.97	0.71
Fatigue	0.94	0.72
Q13	0.97	0.59
Q14	0.96	0.78
Emotion	0.93	0.72
Q15	0.96	0.66
Q16	0.96	0.69
Parental burden	0.94	0.89
Q17	0.96	0.74
Q18	0.96	0.87
Q19	0.96	0.86
Q20	0.96	0.73
Q21	0.96	0.68
Financial burden	0.94	0.67
Q22	0.96	0.68
Satisfaction	0.94	0.77
Q23	0.96	0.77
Q24	0.96	0.78

## Test–retest reliability

Intraclass correlation coefficient (ICC) ranged from 0.70 (Transfer) to 0.96 (Physical function, Daily living, Parental burden) indicating good to excellent agreement.

## Floor and ceiling effects

In the congenital group, the majority of domains leaned towards a right-skewed distribution, with the exceptions of Daily Living, Emotion, and Parental Burden, which were left-skewed. In the idiopathic group, the data mostly exhibited right skewness, aside from the domains of Fatigue and Emotion, which were left-skewed. In the neuromuscular group, Pain and Discomfort, Pulmonary Function, Fatigue, and Emotion lean towards a left skewness while other domains were skewed to the right. In the syndromic group, most domains were right-skewed, with General Health and Fatigue being skewed to the left.

There was a floor effect ranging from 0 to 32% and a ceiling effect ranging from 0 to 77% in the data (Table 2). The presence of floor and ceiling effect was analyzed for each etiology, with the highest rate of ceiling effect being in the Pulmonary Function domain in congenital (77%), idiopathic (77%), and syndromic (63%) groups (Table 3). Patients with neuromuscular scoliosis had the highest floor effect in Transfer and Daily Living (32%) and the highest ceiling effect in Financial Burden (44%).

**Table 3:** Domain and item characteristics

Item	Mean	SD	Median	Floor effect (%)	Ceiling effect (%)	Missing items (%)
General health	69.0	22.0	75	0.0	14.7	
Q1	3.8	0.99	1	0.0	30.3	0.0
Q2	3.8	0.93	4	3.7	20.2	0.0
Pain and discomfort	73.0	23.0	75	0.0	30.3	
Q3	4.0	0.91	4	0.0	32.1	0.0
Q4	4.0	0.94	4	0.0	33.0	0.0
Pulmonary function	90.0	18.0	100	0.9	68.8	
Q5	4.7	0.82	4	0.9	81.7	0.0
Q6	4.7	0.77	5	1.8	78.9	0.0
Transfer	75.0	35.0	100	8.3	58.7	
Q7	3.9	1.46	5	11.0	56.9	0.0
Physical function	76.0	33.0	91.7	6.4	45.0	
Q8	4.2	1.24	5	6.4	60.6	0.0
Q9	4.1	1.47	5	12.8	67.0	0.0
Q10	3.9	1.47	5	13.8	55.0	0.0
Daily living	67.0	38.0	87.5	10.1	45.9	
Q11	3.8	1.55	5	15.6	51.4	1.0
Q12	3.6	1.76	5	24.8	56.9	0.0
Fatigue	74.0	25.0	75	0.9	29.4	
Q13	4.0	0.92	4	0.9	35.8	1.0
Q14	3.9	1.18	4	4.6	39.4	1.0
Emotion	80.0	24.0	87.5	0.9	42.2	
Q15	4.3	0.96	5	0.9	56.0	1.0
Q16	4.1	0.99	4	1.8	45.0	1.0
Parental burden	73.0	26.0	82.5	0.9	17.4	
Q17	3.8	1.17	4	6.4	32.1	1.0
Q18	4.0	1.38	5	10.1	53.2	1.0
Q19	3.8	1.36	4	10.1	43.1	1.0
Q20	4.0	1.04	4	1.8	43.1	1.0
Q21	4.2	1.17	5	4.6	55.0	2.0
Financial burden	82.0	28.0	100	4.6	59.6	
Q22	4.3	1.09	5	4.6	59.6	2.0
Satisfaction	78.0	25.0	82.5	2.8	43.1	
Q23	4.1	0.99	4	2.8	43.1	2.0
Q24	4.2	1.01	5	1.9	46.3	2.0

## Discriminative validity

All other domains except Pain and Discomfort and Pulmonary Function showed statistically significant difference in the Kruskal–Wallis test (Table 3). When etiologies were compared group by group using Dunn's test adjusted for multiple comparisons, there was a statistically significant difference between idiopathic and neuromuscular groups in General Health, Transfer, Physical Function, Daily Living, Fatigue, Emotion, Parental Burden, and Satisfaction domains. There were also statistically significant differences in Physical Function between congenital and neuromuscular groups, Daily Living between congenital and neuromuscular as well as idiopathic and syndromic groups, Emotion between congenital and neuromuscular, Parental Burden between congenital and neuromuscular as well as idiopathic and syndromic, and in Satisfaction domain between congenital and neuromuscular as well as idiopathic and syndromic groups (Table 4).

**Table 4:** Discriminative analysis between the etiologies

	Congenital					Idiopathic					Neuromuscular					Syndromic					
	Mean	SD	Median	Floor effect	Ceiling effect	Mean	SD	Median	Floor effect	Ceiling effect	Mean	SD	Median	Floor effect	Ceiling effect	Mean	SD	Median	Floor effect	Ceiling effect	Significance
General health	70.6	18.2	75.0	0.0	11.8	75.7	20.4	75.0	0.0	20.8	57.0	24.5	62.5	0.0	8.0	59.4	14.6	62.5	0.0	0.0	0.003
Pain and discomfort	75.7	17.9	75.0	0.0	23.5	76.9	22.3	75.0	0.0	37.7	64.0	25.6	75.0	0.0	16.0	75.0	17.7	75.0	0.0	25.0	0.25
Pulmonary function	91.9	17.7	100.0	0.0	76.5	93.9	13.6	100.0	0.0	77.4	83.0	24.7	87.5	4.0	48.0	85.9	20.5	100.0	0.0	62.5	0.063
Transfer	83.8	23.3	100.0	0.0	58.8	85.8	25.7	100.0	0.0	71.7	49.0	43.6	50.0	32.0	36.0	62.5	37.8	62.5	12.5	37.5	< 0.001
Physical function	85.3	19.2	91.2	0.0	35.3	90.6	17.7	100.0	0.0	64.2	42.3	39.7	25.0	28.0	12.0	68.8	33.0	83.3	0.0	25.0	< 0.001
Daily living	67.7	33.1	75.0	5.9	35.3	85.1	29.9	100.0	1.9	75.5	34.0	32.8	25.0	32.0	4.0	42.2	39.5	25.0	12.5	12.5	< 0.001
Fatigue	72.7	22.0	75.0	0.0	23.5	80.7	20.9	87.5	0.0	35.8	58.5	29.5	62.5	4.0	16.0	81.3	18.9	81.3	0.0	37.5	0.008
Emotion	93.4	14.1	100.0	0.0	76.5	82.4	21.5	87.5	0.0	43.4	64.5	26.9	62.5	4.0	16.0	78.1	19.8	75.0	0.0	37.5	< 0.001
Parental burden	81.3	15.6	85.0	0.0	11.8	83.6	20.3	90.0	0.0	24.5	51.2	29.0	45.0	4.0	8.0	78.1	24.6	65.0	0.0	0.0	< 0.001
Financial burden	92.2	15.1	100.0	0.0	70.6	85.6	25.0	100.0	3.8	66.0	69.0	35.6	75.0	12.0	44.0	78.1	24.8	75.0	0.0	37.5	0.043
Satisfaction	86.7	14.3	87.5	0.0	47.1	87.5	20.7	100.0	0.0	62.3	61.5	23.3	62.5	8.0	22.0	67.2	26.6	68.8	0.0	12.5	< 0.001

Major curve was significantly negatively correlated with all domains of the EOSQ-24 except the financial burden in patients with idiopathic scoliosis (correlation efficient ranged between − 0.53 and − 0.27). In the neuromuscular group, the major curve was significantly negatively correlated with domains Pain and Discomfort (*r* = − 0.44, *p* = 0.025), Pulmonary Function (*r* = − 0.65, *p* < 0.001), Physical Function (*r* = − 0.48, *p* = 0.016), Fatigue (*r* = − 0.49, *p* = 0.013), Emotion (*r* = − 0.55, *p* = 0.004), and Financial Burden (*r* = −0.50, *p* = 0.012). Similar, nonsignificant, correlations were seen in congenital and syndromic groups.

## Discussion

Our study presented good internal consistency, test–retest reliability, and discriminative validity of the Finnish translation of EOSQ-24. There was a marked ceiling effect present.

As reported by previous studies, assessing the health-related quality of life in patients with EOS is complicated due to the heterogenous patient population. Patients with neuromuscular and syndromic early onset scoliosis have lower HRQoL scores when compared to patients with idiopathic and congenital scoliosis [8, 9]. Our study shows similar results with patients with idiopathic and congenital scoliosis having higher scores in the EOSQ-24 domains than patients with neuromuscular or syndromic scoliosis.

Previous studies have reported good internal consistency of EOSQ-24 translations [7, 10–21]. Our results were similar with good internal consistency of the domains and the 24 items. Like previous studies, test–retest reliability was satisfactory in our study [7, 15, 18].

Previous studies have reported marked floor and ceiling effects in the EOSQ-24 domains [10, 13, 16, 17, 20, 21]. In the present study, there was a marked floor effect in neuromuscular group in Transfer, Physical Function, and Daily Living domains, indicating that in these domains a marked proportion of the patients answered the worst possible outcome. Ceiling effect refers to the proportion of the patients who reported highest possible scores. There was a marked ceiling effect regardless of the etiology in the Financial Burden domain. The Financial Burden consist of one item #22 “How much of a financial burden has your child’s diagnosis of Early Onset Scoliosis been?”, with most favorable answer option being “No burden”. In Finland, the healthcare is generally free, and the patients and families can receive financial support. There was a marked ceiling effect in all domains in the idiopathic group and most of the domains in the congenital group. Along with high mean and median values, this indicates that the patients with idiopathic and congenital scoliosis generally reported very high HRQL outcomes. The presence of ceiling effects was significantly lower in the neuromuscular and syndromic groups. Patients in the neuromuscular group had the highest presence of the floor and lowest presence of the ceiling effect along with lowest mean scores for the domains, indicating lower HRQL than in other etiologies. The presence of floor and ceiling effects should be considered as a potential source of error during statistical analysis, especially when authors would conclude no difference between treatment groups when a marked floor or ceiling effect would be present [25].

Similar to previous studies, there was a significant negative correlation between the major curve and EOSQ-24 scores in the idiopathic group and some of the domains in the neuromuscular group [11–13, 15, 19, 20]. This indicates that the patients in these groups generally experience decreased quality of life as the spinal deformity worsens. There were similar correlations in the syndromic and congenital group, but these correlations were not statistically significant. This may be due to the restricted sample size of the syndromic and congenital groups.

The translation of EOSQ-24 was successful, and the translated version of the EOSQ-24 was found to be valid and reliable measure. The internal consistency, test–retest reliability, and discriminative abilities were satisfactory. There was a marked ceiling effect present, which should be considered as a potential source of error.

## Data Availability

Data is available from the corresponding author for a reasonable request.
